# Detection of *Bartonella* spp. in foxes' populations in Piedmont and Aosta Valley (NW Italy) coupling geospatially-based techniques

**DOI:** 10.3389/fvets.2024.1388440

**Published:** 2025-01-21

**Authors:** Annalisa Viani, Tommaso Orusa, Sara Divari, Stella Lovisolo, Stefania Zanet, Riccardo Orusa, Enrico Borgogno-Mondino, Enrico Bollo

**Affiliations:** ^1^Azienda USL della Valle d'Aosta, S.C. Sanità Animale, Quart, Italy; ^2^GEO4Agri DISAFA Laboratory, Department of Agricultural, Forest and Food Sciences (DISAFA), University of Turin, Grugliasco, Italy; ^3^Department of Veterinary Sciences, University of Turin, Grugliasco, Italy; ^4^Experimental Zooprophilactic Institute of Piedmont, Liguria and Aosta Valley (IZS PLV) S.C Valle d'Aosta—CeRMAS (National Reference Center for Wildlife Diseases), Quart, Italy

**Keywords:** *Bartonella* spp., fox, public health, epidemiology, GIS and remote sensing

## Abstract

*Bartonella* is a genus of bacteria known to cause various rare but potentially dangerous diseases in humans and wildlife. The objective of this study was to investigate the presence of *Bartonella* spp. in red foxes (*Vulpes vulpes*) from Piedmont and Aosta Valley (NW Italy) and to explore the potential association between environmental humidity and *Bartonella* infection using remote sensing data. A total of 114 spleen samples were collected from hunted red foxes and screened for *Bartonella* spp. DNA using a qPCR assay targeting the *ssrA* locus. Samples that tested positive were further analyzed using end-point PCR to detect the *ssrA, gltA*, and *rpoB* loci. The overall prevalence of *Bartonella* spp. infection was found to be 7.9% (9/114), with a prevalence of 6.9% (7/101) in foxes from Piedmont and 15.4% (2/13) in foxes from Aosta Valley. Sequencing results identified *Bartonella schoenbuchensis* R1 as the most commonly isolated species (5/9, 62.5%), followed by Candidatus “*Bartonella gerbillinarum*” in two samples (2/9, 28.6%). To investigate the relationship between environmental factors and *Bartonella* infection, data from NASA USGS Landsat missions (TOA collection) from 2011 to 2022 were processed using Google Earth Engine. The Tasseled Cap Wetness Index (TCW), an indicator of landscape moisture, was calculated for each meteorological season. The study found that *Bartonella* spp. infections in foxes were positively associated with higher TCW values (>0.7). Canonical Correspondence Analysis further demonstrated a strong link between pathogen prevalence and municipal-level TCW, suggesting that TCW could be used as a parameter to facilitate disease management and control. This study provides a starting point for a more comprehensive epidemiological assessment of *Bartonella* spp. on a national scale and highlights the potential role of environmental humidity in influencing pathogen distribution.

## 1 Introduction

The widespread distribution of the fox (*Vulpes vulpes* Linnaeus) on the Italian territory has resulted in the conduct of multiple studies on the diseases of this species. Over time, some of these diseases have attracted great interest from the scientific world (1, 2), while less attention has been paid to others, such as bartollenosis. Few studies on the presence and spread of this infection in foxes have been published worldwide (3–5); moreover, among the wide range of hosts of this parasite, the epidemiology of bartonellosis in foxes is one of the least investigated. It is well-known that the knowledge and control of animal diseases contributes significantly to the protection of public health, and the health of free-ranging wildlife can act as sensitive sentinel or bio-indicator for broader health issues (6).

*Bartonella* infection in foxes has been described in different part of the world (3–5, 7–13), and it has been reported in wolves and foxes in Spain and other European countries such as Italy (14), Lithuania (15), and Slovakia (16). Since both the wolf and the red fox can come in contact with domesticated animals, especially dogs, they may play a crucial role in the ecology and spread of *Bartonella* (17). In Italy, *Bartonella bovis* and *Bartonella chomelii* are the only species reported in wildlife, mostly in deer ticks (18). In particular, there is a lack of data on infection in wild canids, although *Bartonella vinsonii* subsp. *berkhoffii* and *Candidatus Bartonella merieuxii* have been reported in hunting dogs in southern Italy (19). It is worth to note that *B. vinsonii* subsp. *berkhofii* genotype III can be highly pathogenic to dogs (20).

*Bartonella* spp. is widespread worldwide, but in some areas the prevalence is higher in relation to the climatic conditions that favor the spread of arthropod vectors (11). Moreover, the distribution of *Bartonella* is partly linked to the variety of blood-sucking vectors (12). Transmission occurs indirectly, by means of arthropod vectors, mainly fleas, lice, ticks, and blood-sucking flies, which ingest erythrocytes containing bacterial cells through a blood meal and then transmit them to a reservoir or to an accidental host (13). In the study conducted by de Paiva Diniz et al. (68) in 2018, *Bartonella* strains were identified from environmental and arthropod samples. Furthermore, investigations on the gut microbiome of a variety of insects have revealed the presence of the microorganism among arthropods, i.e., cockroaches, butterflies, bees, various ant species, and a wide variety of ectoparasitic species (15, 16). Other researchers have hypothesized that *Bartonella* may also have a commensal role in arthropods (17), and therefore how this microorganism originated as an environmental bacterium and evolved and diversified through contact with arthropod vectors. *Bartonella* represents an ideal pathogen to focus on to evaluate the impact of anthropogenic activity on the distribution models of the main infectious diseases in animals, within the same species and between different species.

The recent exponential development of science and technologies applied to GIS has contributed to the enhancement of epidemiological data analysis capabilities and has provided new and powerful tools for animal disease surveillance (11). GIS, spatial analysis techniques and the use of satellites provide useful methods for collecting and managing the information necessary for epidemiological studies. Still little explored in the veterinary field is the use and development of applications and methodologies based on “Earth Observation Data”. Within the European space program “Copernicus” and other historical programs such as the NASA Landsat missions, geospatial data with medium-high geometric and temporal resolution allow to exploit and expand risk analysis techniques, translating them into a transfer technology for veterinary studies. To date, in fact, only a few applications have been explored with the use of medium-low resolution data in the context of the NASA “Terra” and “Aqua” missions with the “Modis” sensors (21). Although promising, it should be considered that for eco-epidemiological modeling studies, especially in the Alpine area, these data have strong limitations (22–25). Therefore, to avoid bias related to image processing or to a lack of knowledge of passive satellite remote sensing sensors, it is necessary to have high skills in the field of geomatics and remote sensing (26–28).

The aim of this study was to investigate the prevalence and distribution of *Bartonella* spp., a genus of bacteria responsible for rare but serious diseases, within red fox populations in the provinces of Cuneo and Biella (Piedmont) and Aosta Valley in northwestern Italy. Specifically, the research sought to understand the geographical and environmental factors that might influence the presence of *Bartonella* spp. at the municipal level. To achieve this, the study employed a combination of molecular diagnostic techniques to detect *Bartonella* DNA in fox spleen samples and advanced satellite remote sensing to analyze environmental conditions. By integrating these approaches, the research aimed to explore possible correlations between the prevalence of *Bartonella* infections and environmental factors, particularly focusing on landscape moisture as indicated by the Tasseled Cap Wetness Index (TCW). The ultimate objective was to determine whether specific environmental conditions, such as higher humidity levels, could be associated with increased *Bartonella* infection rates in fox populations. This interdisciplinary approach aimed to provide a more comprehensive understanding of the epidemiology of *Bartonella* spp. in wildlife and contribute valuable insights for disease management and control efforts on a broader scale. The study period involved data from 2011 to 2022, during which satellite imagery was processed to create composite TCW images aggregated for each meteorological season of every year.

## 2 Materials

The foxes examined in this study belong to the species *V. vulpes*. A total of 114 spleen samples were collected from foxes either hunted during the hunting seasons or found dead across different locations in the Piedmont districts of the provinces of Cuneo and Biella, as well as the Aosta Valley in northwestern Italy. These samples were collected over a period from 2011 to 2022, with the majority coming from Biella (57 samples), followed by Cuneo (44 samples), and Aosta Valley (13 samples). The samples were stored at −20°C until DNA extraction. Detailed information on the location, sex (59 males and 55 females), age (87 adults, 13 subadults, 14 juveniles), and season of collection (88 in autumn-winter, 26 in spring-summer) is summarized in Supplementary Tables S1a–c.

### 2.1 Data collection

#### 2.1.1 Lab data

For the detection of *Bartonella* spp. DNA, ~30 mg of spleen tissue was thawed and subjected to DNA extraction using the “E.Z.N.A. Tissue DNA Kit” (Omega Bio-Tek, Norcross, USA), following the manufacturer's instructions. The CFX Connect Real-Time System (BioRad, Hercules, USA) was used to perform qPCR targeting the *ssrA* locus, which codes for transfer-messenger RNA. Samples with a Cq value lower than 32 were considered positive for *Bartonella* spp. Positive samples underwent further analysis using end-point PCR to detect the *gltA, rpoB*, and *ssrA* loci. Detailed protocols for each PCR assay are provided, including the conditions for amplification and subsequent sequencing of the PCR products. The end-point PCR products were separated, purified, and sent for bidirectional DNA Sanger sequencing.

#### 2.1.2 GIS data

The study utilized multispectral optical remote sensing data from the NASA USGS Landsat 5 and Landsat 8 missions. The data was processed using the cloud-based platform Google Earth Engine (GEE), which allows for large-scale processing of satellite images. The study specifically focused on the Tasseled Cap Wetness (TCW) index, a multispectral index related to soil and canopy moisture interactions, computed from Landsat data. Composite images were created for each meteorological season from 2011 to 2022, aggregating data to capture seasonal variations in environmental conditions. The TCW index was calculated for each scene to assess the relationship between landscape moisture and the prevalence of *Bartonella* spp. in foxes. The Moran's I index was used to test for spatial autocorrelation.

## 3 Methods

The approach reported below in each sub-chapter was based on the following materials.

### 3.1 DNA extraction and qPCR

The CFX Connect Real-Time System (BioRad, Hercules, USA) was used for qPCR and the locus taken into consideration was *ssrA* codifying for transfer-messenger RNA (29). The protocol includes an initial denaturation at 95°C for 3 min, followed by 45 cycles of denaturation (at 95°C for 15 s), hybridization and extension (at 60°C for 1 min). qPCR was performed using IQ Multiplex Powermix (BioRad, Hercules, USA).

The genomic DNA of *Bartonella* spp. FG4-1 was used as a positive control (30). Samples with a Cq value lower than 32 were considered positive and were subjected to subsequent analysis.

Primers and probe related to the loci considered in this study are reported in Supplementary Table S2.

### 3.2 PCR end-point of *gltA, rpoB* and *ssrA* loci

The citrate synthase (*gltA*), RNA polymerase (*rpoB*), and *ssrA* loci were detected in qPCR positive samples using end-point PCR. For the *gltA* locus, two PCR end-point assays were applied, generating 380 bp (31) and 700 bp (32) amplicons. Amplification was performed using a T100 Thermal Cycler (BioRad, Hercules, USA). The protocols applied in the end-point PCR were the following:

- *gltA* (380 bp): an initial denaturation at 95°C for 15 min followed by 35 cycles at 95°C for 20 s, at 51°C for 30 s and at 72°C for 2 min;
- *gltA* (700 bp): an initial denaturation at 95°C for 15 min followed by 40 cycles at 95°C for 30 s, at 48°C for 1 min, at 72°C for 1 min; finally, an extension at 72°C for 7 min;
- *rpoB*: an initial denaturation at 95°C for 15 min followed by 35 cycles at 95°C for 30 s, at 53°C 78 for 30 s, at 72°C for 1 min; finally, an extension at 72°C for 2 min;
- *ssrA*: an initial denaturation at 95°C for 30 s followed by 40 cycles at 95°C for 15 s, at 60°C for 1 min, at 72°C for 30 s; finally, an extension at 72°C for 3 min.

Reactions were performed using HotStartTaq matrer mix (Qiagen) using primers described in Supplementary Table S2.

End-point PCR products were separated on 1.5% agarose gel in Tris-Acetate-EDTA (TAE) buffer using MIDORI Green Advance DNA stain (Nippon Genetics, Düren, Germany).

### 3.3 Sequencing

The amplicons obtained were purified using MinElute PCR Purification Kit (Qiagen, Hilden, Germany) and they were sent to BMR Genomics (Padua, Italy) for bidirectional sequencing (DNA Sanger sequencing) (BMR Genomics, Padua, Italy). Sequences obtained were manually corrected using the Geneious Prime 2020.1. The corrected sequences were aligned with the sequences deposited in GenBank, by BLAST (https://blast.ncbi.nlm.nih.gov/Blast.cgi, last access on 19 April 2023).

### 3.4 Statistic analysis

The results obtained were analyzed using the GraphPad Prism 8.4.2 software (GraphPad Software, California, USA). Fisher's test was used to evaluate the association between the presence of *Bartonella* spp. and gender, age, season, and period of collection of samples. The Chi square test was used to evaluate the association between the presence of *Bartonella* spp. and the place of recovery of animals. A 95% confidence interval was set, and results with *p*-value < 0.05 were considered statistically significant.

### 3.5 GIS and remote sensing

The GIS analyzes were carried out using the software QGIS version 3.16.4 Hannover (23). For the geospatial analysis, we used multispectral optical remote sensing data from the NASA USGS Landsat missions with 16-day time resolution starting from 1972 for the whole globe and spatial resolution as a function of the considered spectral band of 30 m for the most part of the spectral bands used. For this work, data from the USGS Landsat 5 and USGS Landsat 8 missions were used, respectively from the Multispectral Scanner (MSS) and Operational Land Imager (OLI) sensors. In particular, the cloud-based platform Google Earth Engine (hereinafter called as GEE) was adopted for the processing of remote sensing data, which allows large-scale processing of satellite images to detect changes, map trends and quantify differences on the Earth's surface (33–40) by accessing petabytes of various satellite missions from different global space agencies.

In particular, the following GEE collections were used:

- LANDSAT/LT05/C02/T1_TOA: Landsat 5 MSS TM with reflectance calibrated at the level of the upper part of the atmosphere (TOA). Calibration coefficients are extracted from each scene's metadata for TOA calculation based on (41).
- LANDSAT/LC08/C02/T1_TOA: Landsat 8 OLI TIRS with reflectance calibrated at the level of the upper part of the atmosphere (TOA). Calibration coefficients are extracted from each scene's metadata for TOA calculation based on (41, 42).

Starting from the sample collection data of the entire population of foxes considered, aggregated by meteorological seasons (43) and related year using a GEO4Agri DISAFA Lab script, average composite images were created for each meteorological season (Supplementary Table S5) starting from the year 2011 until summer 2022 (Supplementary Tables S3, S4).

From the empirical evidence reported in the literature (44, 45), in which the pathogen appears to be influenced by humidity, for each scene from which the composites were obtained the multispectral TCW index was computed.

The Tasseled Cap (46) was generated based on spectral information from the Landsat satellite. The Tasseled Cap coefficients used in the linear equation of the Tasseled Cap transformation are sensor specific and therefore have been derived for each sensor system. The index is related to principal component analysis and vegetation indices. In this case reference was made only to the TCW, a multispectral index of humidity. The Tasseled Cap Wetness provides a measured value for soil and canopy moisture interactions, highlighting how moisture is distributed between the soil and the plant canopy.


$$\begin{array}{l} TCW=\left(\rho_{blue}*\alpha_{1}\right)+\left(\rho_{green}*\alpha_{2}\right)+\left(\rho_{red}*\alpha_{3}\right)\\ \;+\left(\rho_{NIR}*\alpha_{4}\right)+\left(\rho_{SWIR1}*\alpha_{6}\right)+\left(\rho_{SWIR2}*\alpha_{7}\right) \end{array}$$

where:

ρ = TOA reflectance corresponding to the regions of the electromagnetic spectrum of interest.

α = coefficient for the specific TCW transformation for each Landsat band 5 and 8, respectively. Reference was made to (47, 48).

To demonstrate a possible relationship between the environmental components TCW mappable by remote sensing and municipal-based positivity, Moran's index I was calculated to test the existence of a spatial problem, which in all cases was higher than Moran *I* > 0.90, consequently significant:


$$\begin{array}{l} I=\frac{N}{\Sigma i\Sigma jW_{ij}}\frac{\Sigma y\Sigma jxW_{ij}\left(x_{i}-u\right)}{\Sigma i\left(x_{i}-u\right)} \end{array}$$

where:

- *N* = is the number of geographical units;

- x_i_ = is the variable that describes the phenomenon under study in region i;

- μ = represents the sample mean and therefore (xi-μ) is the deviation from the mean of the variable of interest;

- W_ij_ = is the weight matrix which in many cases is equivalent to a binary matrix i, j in which weights are used that are inversely proportional to the distance between point i and point j (where “i” is different from “j”);

- I = varies between −1.0 and +1.0 and its numerator is interpreted as the covariance between contiguous units.

## 4 Results

### 4.1 qPCR and PCR end-point

All 114 selected animals were analyzed by qPCR for the presence of the *ssrA* locus. Nine out of 114 (7.9 %) samples were positive by qPCR analysis and, in particular, 7/101 (6.9%) and 2/13 (15.4%) in red foxes collected in Piedmont and Aosta Valley, respectively. The positive samples further analyzed with end-point PCR yielded 7/9 samples positive for the *ssrA* locus, of which 3/9 positive for the *gltA* locus (380 and 700 bp) and none for the *rpoB* locus. Two sequences were not evaluable due to low quality.

In Table 1 sequencing results of *Bartonella* spp. positive foxes are reported. The closest relative sequence and identity results for *ssrA* and *gltA* are described.

**Table 1:** Sequencing results of *Bartonella* spp. positive foxes, including the closest relative sequence (query cover 97–100%) and identity results (ID %) for *ssrA* and *gltA* loci, number (n.), and origin of finding.

**No. of samples**	**Sample ID**	**Origin**	**Closest relative DNA sequences (Accession No.)**			
			***ssrA***	**ID %**	***gltA* (380 bp, 700 bp)**	**ID %**
2	255/13	Sandigliano (BI)	Candidatus “*Bartonella gerbillinarum*” (MH618860.1)	97.63	ND	
	339/14	Vigliano (BI)	Candidatus “*Bartonella gerbillinarum*” (MH618860.1)	98.51	ND	
5	34/20	Monterosso Grana (CN)	*Bartonella schoenbuchensis* R1 (CP019789.1)	100	*Bartonella schoenbuchensis* R1 (CP019789.1)	100
	243/13	Salussola (BI)	*Bartonella schoenbuchensis* R1 (CP019789.1)	97	*Bartonella schoenbuchensis* R1 (CP019789.1)	99.7–99.3
	362/14	Biella	*Bartonella schoenbuchensis* R1 (CP019789.1)	100	*Bartonella schoenbuchensis* R1 (CP019789.1)	100
	21/21	Dronero (CN)	*Bartonella schoenbuchensis* R1 (CP019789.1)	100	ND	
	486/12	Mosso Santa Maria (BI)	*Bartonella schoenbuchensis* R1 (CP019789.1)	99.6	ND	

Based on the sequencing of the amplicons related to the *ssrA* locus, the most represented *Bartonella* species was *B. schoenbuchensis* R1 (5/9, 55.6%); Candidatus “*Bartonella gerbillinarum*” was identified in two animals (2/9, 22.2%). *B. schoenbuchensis* R1 was also confirmed by sequencing the *gltA* locus in 3/9 animals.

### 4.2 Data distribution

No statistically significant associations were observed between the presence of *Bartonella* spp. and the sex, age of the foxes, animal area (province), years (period) and seasons. Further information regarding statistical tests specifically: *F*-test, *t*-test, Fligner-Kileen test for equal coefficients of variation and Chi-Square can be found in Supplementary material.

### 4.3 Geostatistical analysis

In order to analyze a possible relationship between TCW and the presence of *Bartonella* spp. for the composite timeseries on a seasonal basis for the years of interest, zonal statics were created: in this case Spatial Join on a municipal basis for the Aosta Valley and for the provinces of Biella and Cuneo, respectively. The municipal-based and non-punctual analysis was dictated by the fact that the samples were not punctually geo-referenced and starting from the assumption that the place where the animal was found (the municipality and neighboring area) is its core home range (moreover since we are dealing with foxes, the assumption finds correspondence in the ecology and ethology of this species).

**Figure 1:**
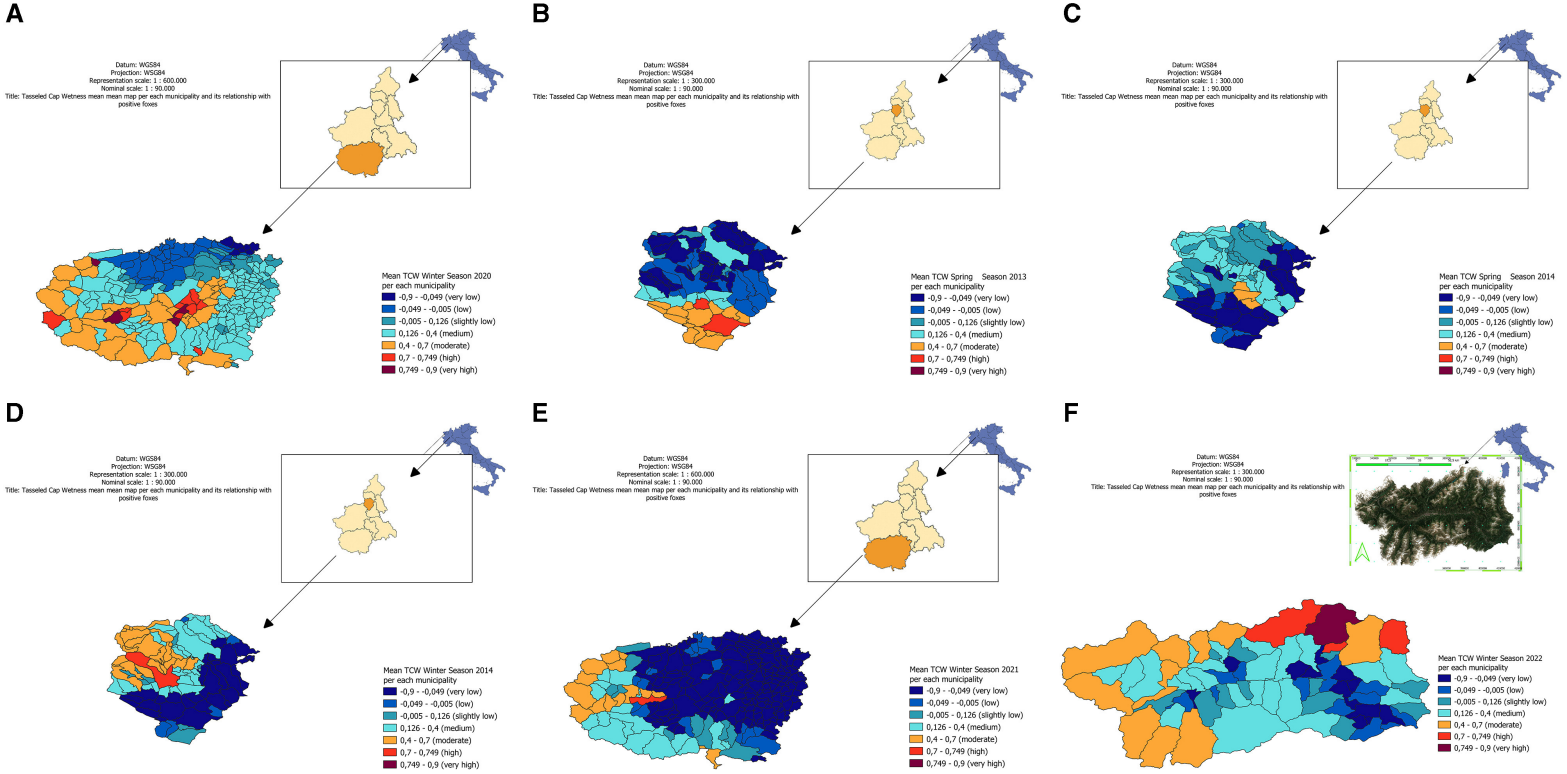
Local spatial autocorrelation indicator (LISA) map for Tasseled Cap Wetness (TCW) for each municipality, in the different periods analyzed. **(A, E)** in Piedmont, province of Cuneo. **(B–D)** in Piedmont, province of Biella. **(F)** in Aosta Valley region.

In order to map the risk of distribution based on geostatistical results obtained, LISA maps (Local Spatial Autocorrelation Indicators) were created on a municipal scale for the periods of interest [Figure 1 reports LISA map for winter season in (Piedmont province of Cuneo) during the year 2020]. The other LISA maps are attached in Supplementary material. In particular, figure A reports LISA map for spring season in Piedmont (province of Biella) during the year 2013, figure B the spring season in Piedmont (province of Biella) during the year 2014, figure C the winter season in Piedmont (province of Biella) during the year 2014, figure D the winter season in Piedmont (province of Cuneo) during the year 2021, and finally figure E the winter season in Aosta Valley during the year 2022. Specifically, in all the maps (Figures 1A–E), warm colors (dark red, red, orange) pointed out a strong correlation between samples and TCW, while cold colors (dark blue, blue, green) a low correlation. It's worth to note that in areas where low correlations occur there were no positive samples. Due to the strong spatial correlation observed between TCW and the presence of *Bartonella* spp., a threshold of 0.7 TCW was identified as a potential minimum limit range to detect the pathogen, as determined by the Moran's I index.

## 5 Discussion

The activity carried out in this study provides an interesting contribution to the knowledge of bartonellosis in the fox populations of Piedmont and Aosta Valley, starting from the year 2011 until today. First, the results show how this pathology is present in the area with rather low prevalence values, equal to 7.9% (nine positive samples out of a total of 114). Molecular investigations have highlighted the presence of *Bartonella* DNA belonging to the species *B. schoenbuchensis* R1 (sequence identity of 99.6–100%) in five foxes, two from the province of Cuneo and three from the province of Biella. This species of *Bartonella* causes bacteremia in ruminants and has been isolated from bloodsucking lice such as *Lipoptena cervi* which affect deer, roe deer, moose and sika in Europe, Siberia, and northern China, as well as red deer, moose, horses, and white-tailed cattle in North America. Accidental human infestation with *Lipoptena cervi* is well-documented.

Candidatus “*Bartonella gerbillinarum*” DNA was identified in two animals with a sequence identity of 97.6–98.5%. Moreover, for this species, several insect vectors seem to play an important role in its spread, the microbiological evaluation and epidemiological significance of which require further studies (49, 50).

Statistical analysis of the data did not reveal a statistically significant association in the distribution of data between season, year, age, sex, and municipality of origin of the samples. This result can be attributed to the scarcity of positive animals and to a rather homogeneous distribution of positivity in relation to the above factors mentioned. In fact, previous studies conducted in other species (51) have showed a different prevalence of *Bartonella* in summer instead of in autumn. Moreover, the prevalence was high in juvenile and subadults and declined considerably in largest-oldest individuals.

The weather and environment affect rat infections, including the ecology of zoonotic pathogens in urban ecosystems (45): high minimum temperatures were positively correlated with *Bartonella tribocorum* infection in rats for several time periods before the rats were captured. This result implies that *B. tribocorum* transmission among rats and flea vector survival may require a baseline minimum temperature.

The trend of the infection during the year is certainly also linked to the presence of the vector, which strictly depends on the environmental conditions, which can vary greatly in different seasons. Regarding fleas, it must be considered that the immature stages complete their life outside the host and consequently depend more than adults on environmental conditions (52). Humidity seems to play a fundamental role in the survival of fleas: larvae of *Xenopsylla conformis* and *X. ramesis* are more sensitive to low humidity conditions, as they are unable to close their spiracles and consequently prevent water loss through the respiratory tract (52). It's worth to note that some environmental factors are necessary for arthropod-borne infections in order to promote the development of suitable vectors, and infectious agents within these vectors (53). An increasing number of arthropods could act as potential *Bartonella* species vectors and, for this reason, they should be taken into consideration on clinical and serological data from human and canine cases. In particular, arthropod vectors that spend a large part of their life cycle away from their hosts, like ticks and mosquitoes, are predicted to have strict habitat requirements, whereas lice and fleas, which are always on their hosts or in their shelters, typically have more flexible requirements (54, 55).

As regards the geostatistical analysis, the TCW seems to be a good environmental indicator for identifying the presence of conditions favorable to the presence of *Bartonella* spp. It is interesting to note that based on the analysis conducted in the years of positivity of the tested samples, the TCW is >0.7 in the considered municipalities. Therefore, it is possible to establish a relationship between a threshold TCW and *Bartonella* spp. presence. In fact, the seasonal TCW was positively associated with *Bartonella* spp. infection in foxes as infection was always associated to TCW > 0.7. However, this is a preliminary analysis that deserves further insights. In fact, the population analyzed was small and the geographic area was limited. Despite this, the obtained results seem to corroborate the thesis of a close link between *Bartonella* spp. and humidity, demonstrating how ordinary epidemiological monitoring techniques can be associated and supported by remote sensing, for a territorial scale understanding of health phenomena in a perspective of “One Health” and technology transfer (56–61). While humidity may influence vector abundance and survival, its direct correlation with *Bartonella* infection rates may be influenced by a myriad of other factors. Conducting epidemiological studies and vector surveillance in specific geographic regions can provide valuable insights into the relationship between humidity and *Bartonella* transmission dynamics. In particular, due to the fact that Tasseled Cap Wetness is a spectral transformation technique commonly used in remote sensing to capture and quantify surface moisture content, by leveraging satellite imagery and spectral bands sensitive to moisture, this factor can provide valuable insights into environmental conditions conducive to vector proliferation and subsequent pathogen transmission, such as *Bartonella*. In regions where humidity plays a critical role in vector abundance and activity, Tasseled Cap Wetness can serve as a proxy for moisture levels in the environment. Areas exhibiting higher Tasseled Cap Wetness values may indicate regions with elevated humidity, potentially creating favorable conditions for vector breeding and survival. By integrating Tasseled Cap Wetness data with epidemiological records of *Bartonella* infection rates, researchers can explore spatial patterns and temporal trends in *Bartonella* transmission dynamics. Areas characterized by high Tasseled Cap Wetness values could be prioritized for targeted vector surveillance and control efforts, helping to mitigate the risk of *Bartonella* transmission to human and animal populations. Furthermore, the incorporation of Tasseled Cap Wetness into predictive model frameworks can enhance the accuracy and reliability of *Bartonella* risk assessments. By incorporating environmental variables such as humidity, temperature, land cover, and vector habitat suitability, researchers can develop more robust models for forecasting *Bartonella* outbreaks and identifying high-risk areas. For these reasons, the integration of Tasseled Cap Wetness data into studies examining the relationship between humidity and *Bartonella* infection holds great potential for advancing our understanding of vector-borne disease dynamics. In this way, researchers can gain valuable insights into the complex interactions between environmental factors and infectious disease transmission, ultimately informing more effective public health interventions and control strategies (62–65).

Future studies could play a crucial role in advancing our understanding of *Bartonella* spp. on both national and international levels. Expanding research to include a broader range of geographic regions and wildlife hosts would provide a more comprehensive epidemiological map of *Bartonella* distribution. Such efforts could lead to the development of more effective monitoring and control strategies for *Bartonella*-related diseases, which are increasingly recognized as emerging threats to both animal and human health.

Moreover, these studies would not only enhance the field of veterinary medicine by fostering new levels of expertise but also encourage greater collaboration across disciplines, including ecology, epidemiology, molecular biology, and public health. By promoting an interdisciplinary approach, researchers can better address the complexities of pathogen-host-environment interactions. This is especially important in the context of the “One Health” framework, which emphasizes the interconnectedness of human, animal, and environmental health.

The integration of advanced technologies, such as remote sensing, geographic information systems (GIS), and bioinformatics, could facilitate the identification of environmental factors that influence *Bartonella* transmission dynamics. High-resolution data from newer satellite missions, such as the Sentinel-2 or WorldView series or the newer space Italo-European space program IRIDE (66, 67), could be particularly valuable in this context. These missions offer greater spatial and temporal resolution, allowing for more detailed mapping of environmental conditions and finer-scale analysis of habitat characteristics. Such precise data could enhance our ability to detect subtle changes in landscape features that may affect the spread of *Bartonella* spp., enabling more targeted and effective interventions.

In addition, these technological advancements could support the development of predictive models, enabling more proactive disease management and potentially reducing the risk of zoonotic transmission to humans. Ultimately, this research could contribute to the establishment of a global surveillance network for *Bartonella* and other vector-borne pathogens, ensuring a more coordinated and effective response to emerging health threats.

## 6 Conclusions

This study provided a better understanding of the prevalence and genetic diversity of *Bartonella* species in the fox populations of Piedmont and Aosta Valley (NW Italy). Two species of *Bartonella* have been detected in foxes, including *B. schoenbuchensis* R1, responsible of infection in humans. The results raise the potential threats to public health from *Bartonella*: for these reasons, its surveillance in animals and investigations on suspected clinical cases in humans must be strengthened not only in the considered provinces, but also to the rest of the Italian territory, as already demonstrated by the recent studies (14).

## Data Availability

The original contributions presented in the study are included in the article/Supplementary material, further inquiries can be directed to the corresponding author/s.
