# Supplementary material for: Detection of Bartonella spp. in foxes' populations in Piedmont and Aosta Valley (NW Italy) coupling geospatially-based techniques
Source: Front Vet Sci. 2025 Jan 21;11:1388440. doi: 10.3389/fvets.2024.1388440 (PMC11790647; doi:10.3389/fvets.2024.1388440)
Supplement: Supplementary file 1 [file Data_Sheet_1.docx]

Supplementary Material

# Supplementary Data

**Table S1a** List of the samples examined with relative characteristics for the Aosta Valley.

| **ID** | **YEAR** | **SEASON** | **SEX** | **AGE** | **MUNICIPALITY** |
| --- | --- | --- | --- | --- | --- |
| 89300 | 2021 | winter | M | adult | Chatillon |
| 89957 | 2021 | winter | F | subadult | Lillianes |
| 88583 | 2021 | winter | F | adult | Bard |
| 89303 | 2021 | winter | F | adult | Chatillon |
| 92972 | 2021 | winter | M | adult | Brusson |
| 94977 | 2021 | winter | M | adult | Saint-Christophe |
| 89351 | 2021 | winter | M | adult | Arvier |
| 97590 | 2021 | winter | F | adult | Gressoney-Saint-Jean |
| 97350 | 2021 | winter | F | adult | Allein |
| 105939 | 2021 | winter | M | subadult | Aosta |
| 3666 | 2022 | winter | F | subadult | Aosta |
| 1853 | 2022 | winter | F | subadult | Valtournanche |
| 5102 | 2022 | winter | M | adult | Sarre |

**Table S1b** List of the samples examined with relative characteristics for the province of Cuneo (Piedmont).

| **ID** | **YEAR** | **SEASON** | **SEX** | **AGE** | **MUNICIPALITY OF FINDING** |
| --- | --- | --- | --- | --- | --- |
| 19/21 | 2021 | winter | F | adult | Villar San Costanzo |
| 20/21 | 2021 | winter | M | adult | Villar San Costanzo |
| 21/21 | 2021 | winter | M | adult | Dronero |
| 22/21 | 2021 | winter | M | adult | Caraglio |
| 23/21 | 2021 | winter | M | adult | Valgrana |
| 30/21 | 2021 | winter | F | adult | Villar San Costanzo |
| 31/21 | 2021 | winter | M | adult | Villar San Costanzo |
| 15/20 | 2020 | winter | M | adult | Valgrana |
| 17/20 | 2020 | winter | F | adult | Caraglio |
| 24/20 | 2020 | winter | M | adult | Caraglio |
| 31/20 | 2020 | winter | F | adult | Dronero |
| 32/20 | 2020 | winter | M | adult | Dronero |
| 34/20 | 2020 | winter | M | adult | Monterosso Grana |
| 39/20 | 2020 | winter | M | adult | Dronero |
| 40/20 | 2020 | winter | M | adult | Dronero |
| 42/20 | 2020 | winter | F | adult | Bernezzo |
| 43/20 | 2020 | winter | F | adult | Dronero |
| 44/20 | 2020 | winter | F | adult | Cartignano |
| 59/20 | 2020 | winter | F | juvenile | Valgrana |
| 60/20 | 2020 | winter | F | adult | Valgrana |
| 61/20 | 2020 | winter | F | adult | Dronero |
| 518/18 | 2018 | autumn | M | adult | Caraglio |
| 574 bis | 2018 | autumn | F | adult | Caraglio |
| 579/18 | 2018 | autumn | F | adult | Villar San Costanzo |
| 15/16 | 2016 | winter | M | adult | Dronero |
| 16/16 | 2016 | winter | M | adult | Dronero |
| 17/16 | 2016 | winter | M | adult | Dronero |
| 18/16 | 2016 | winter | M | adult | Caraglio |
| 33/16 | 2016 | winter | F | adult | Valgrana |
| 34/16 | 2016 | winter | F | adult | Caraglio |
| 08/22 | 2022 | winter | M | adult | Dronero |
| 09/22 | 2022 | winter | F | adult | Caraglio |
| 10/22 | 2022 | winter | M | juvenile | Caraglio |
| 12/22 | 2022 | winter | M | adult | Caraglio |
| 13/22 | 2022 | winter | M | juvenile | Montemale di Cuneo |
| 14/22 | 2022 | winter | F | adult | Caraglio |
| 15/22 | 2022 | winter | M | juvenile | Caraglio |
| 16/22 | 2022 | winter | F | juvenile | Caraglio |
| 17/22 | 2022 | winter | M | juvenile | Dronero |
| 18/22 | 2022 | winter | M | adult | Caraglio |
| 31/22 | 2022 | winter | M | adult | Monterosso Grana |
| 32/22 | 2022 | winter | F | juvenile | Caraglio |
| 33/22 | 2022 | winter | M | juvenile | Caraglio |
| 35/22 | 2022 | winter | F | adult | Caraglio |
| 36/22 | 2022 | winter | F | juvenile | Valgrana |
| 37/22 | 2022 | winter | F | adult | Monterosso Grana |

**Table S1c** List of the samples examined with relative characteristics for the province of Biella (Piedmont).

| **ID** | **YEAR** | **SEASON** | **SEX** | **AGE** | **MUNICIPALITY OF FINDING** |
| --- | --- | --- | --- | --- | --- |
| 63/21 | 2021 | spring | M | adult | Quaregna |
| 69/21 | 2021 | winter | F | adult | Anzasco |
| 70/21 | 2021 | winter | M | adult | Pratrivero |
| 71/21 | 2021 | winter | M | adult | Miagliano |
| 108/21 | 2021 | winter | F | adult | Cerrione |
| 109/21 | 2021 | winter | F | adult | Camburzano |
| 114/21 | 2021 | winter | F | adult | Mongrando |
| 115/21 | 2021 | winter | F | subadult | Masserano |
| 116/21 | 2021 | winter | F | juvenile | Cavaglià |
| 117/21 | 2021 | winter | M | adult | Crevacuore |
| 132/21 | 2021 | spring | F | adult | Lessona |
| 180/21 | 2021 | summer | F | juvenile | Pollone |
| 70/18 | 2018 | winter | M | juvenile | Cossato |
| 427/18 | 2018 | summer | M | adult | Bocchetta di Margosio |
| 08/17 | 2017 | winter | M | adult | Biella |
| 09/17 | 2017 | winter | M | adult | Vergnasco |
| 53/17 | 2017 | winter | M | adult | Mottalciata |
| 59/17 | 2017 | winter | M | adult | Sagliano |
| 200/14 | 2014 | spring | M | adult | Candelo |
| 203/14 | 2014 | spring | F | adult | Campiglia Cervo |
| 339/14 | 2014 | spring | F | adult | Vigliano |
| 481/14 | 2014 | summer | F | subadult | Massazza |
| 628/14 | 2014 | winter | M | adult | Pollone |
| 362/14 | 2014 | winter | F | adult | Biella |
| 242/13 | 2013 | spring | F | adult | ND* |
| 243/13 | 2013 | spring | M | adult | Salussola |
| 246/14 | 2013 | spring | M | adult | Roasio |
| 247/13 | 2013 | spring | M | subadult | Ponte Strona |
| 248/13 | 2013 | spring | F | adult | Cossato |
| 249/13 | 2013 | spring | F | adult | Veglio |
| 250/13 | 2013 | spring | M | adult | Biella |
| 253/13 | 2013 | spring | M | adult | Crocemosso |
| 254/13 | 2013 | spring | M | adult | Magnonevolo |
| 255/13 | 2013 | spring | F | adult | Sandigliano |
| 485/12 | 2012 | spring | F | subadult | ND* |
| 486/12 | 2012 | spring | M | subadult | Mosso Santa Maria |
| 520/12 | 2012 | spring | M | adult | Pray |
| 785/12 | 2012 | autumn | F | juvenile | Sostegno |
| 83/11 | 2011 | winter | F | adult | Valdengo |
| 112/11 | 2011 | winter | M | adult | Ailoche |
| 113/11 | 2011 | winter | F | juvenile | Pettinengo |
| 114/11 | 2011 | winter | M | adult | Pralungo |
| 119/11 | 2011 | winter | F | subadult | Gaglianico |
| 120/11 | 2011 | winter | M | subadult | Sandigliano |
| 137/11 | 2011 | winter | M | subadult | Cavaglià |
| 138/11 | 2011 | winter | M | juvenile | Occhieppo Superiore |
| 139/11 | 2011 | winter | F | adult | Mongrando |
| 485/11 | 2011 | spring | F | adult | ND* |
| 490/11 | 2011 | spring | F | adult | Tollegno |
| 491/11 | 2011 | spring | F | adult | Biella |
| 495/11 | 2011 | spring | M | juvenile | Pollone |
| 497/11 | 2011 | spring | M | juvenile | ND* |
| 875/11 | 2011 | autumn | M | adult | ND* |
| 881/11 | 2011 | autumn | F | adult | Cossato |
| 908/11 | 2011 | winter | F | adult | Pray |

ND*: not defined. F: female. M: male. Juvenile: 0-4/6 months. Subadult: 1-2 years. Adult: >2 years. Seasons were determined on the basis of the date of collection of the samples according to the calendar of the astronomical seasons.

**Table S2** Primers and probe related to the loci considered in this study (bp: base pairs).

| **Loci** | **ID Primer** | **Sequence 5’-3’** | **bp** | **References** |
| --- | --- | --- | --- | --- |
| *ssrA* | *ssrA*-F | GCTATGGTAATAAATGGACAATGAATAA | 301 | [21] |
|  | *ssrA*-R | GCTTCTGTTGCCAGGTG |  |  |
|  | probe | FAM-ACCCCGCTT AAACCTGCGACG-BHQ1 |  |  |
| *gltA* | 443f | GCTATGTCTGCATTCTATCA | 700 | [24] |
|  | BhCS1137.n | AATGCAAAAAGAACAGTAAACA |  |  |
|  | 781p | GGGGACCAGCTCATGGTGG | 380 | [23] |
|  | BhCS1137.n | AATGCAAAAAGAACAGTAAACA |  |  |
| *rboB* | 1400F | CGCATTGGCTTACTTCGTATG | 825 | [47] |
|  | 2300R | GTAGACTGATTAGAACGCTG |  |  |

**Table S3** X USGS Landsat 5 MSS TM.

| **USGS LANDSAT 5 MSS TM** | | |
| --- | --- | --- |
| **Spectral band** | **Wavelength λ (µm)** | **Description** |
| B1 | 0.45-0.52 | Blue |
| B2 | 0.52-0.60 | Green |
| B3 | 0.63-0.69 | Red |
| B4 | 0.77-0.90 | NIR |
| B5 | 1.55-1.75 | SWIR1 |
| B7 | 2.08-2.35 | SWIR2 |
| QA_PIXEL |  | Pixel quality mask for cloud, shadows and other biophys-ical surfaces |
| QA_RADARSAT |  | Radiometric saturation qual-ity mask |

**Table S4** USGS Landsat 8 OLI TIRS.

| **USGS LANDSAT 8 OLI TIRS** | | |
| --- | --- | --- |
| **Spectral band** | **Wavelength λ (µm)** | **Description** |
| B2 | 0.45-0.51 | Blue |
| B3 | 0.53-0.59 | Green |
| B4 | 0.64-0.67 | Red |
| B5 | 0.85-0.88 | NIR |
| B6 | 1.57-1.65 | SWIR1 |
| B7 | 2.11-2.29 | SWIR2 |
| QA_PIXEL |  | Pixel quality mask for cloud, shadows and other biophysical surfaces |
| QA_RADARSAT |  | Radiometric saturation quality mask |

**Table S5** Distribution of meteorological seasons for each year.

| **Weather season** | **Start (month-day)** | **End (month-day)** |
| --- | --- | --- |
| Autumn | 09-01 | 11-30 |
| Winter | 12-01 | 02-28 |
| Spring | 03-01 | 05-31 |
| Summer | 06-01 | 08-31 |

Figure A. Local spatial autocorrelation indicator (LISA) map for Tasseled Cap Wetness (TCW) for each municipality in Piedmont (province of Biella) in spring 2013.


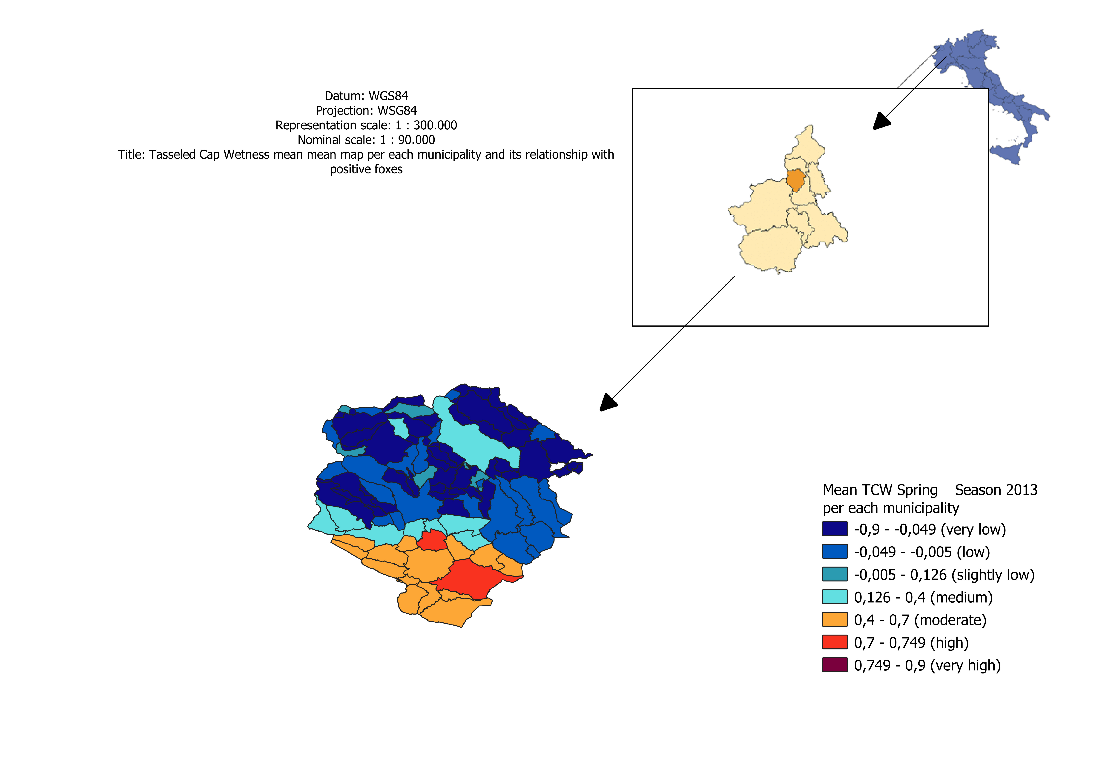


Figure B. Local spatial autocorrelation indicator (LISA) map for Tasseled Cap Wetness (TCW) for each municipality in Piedmont (province of Biella) in spring 2014.


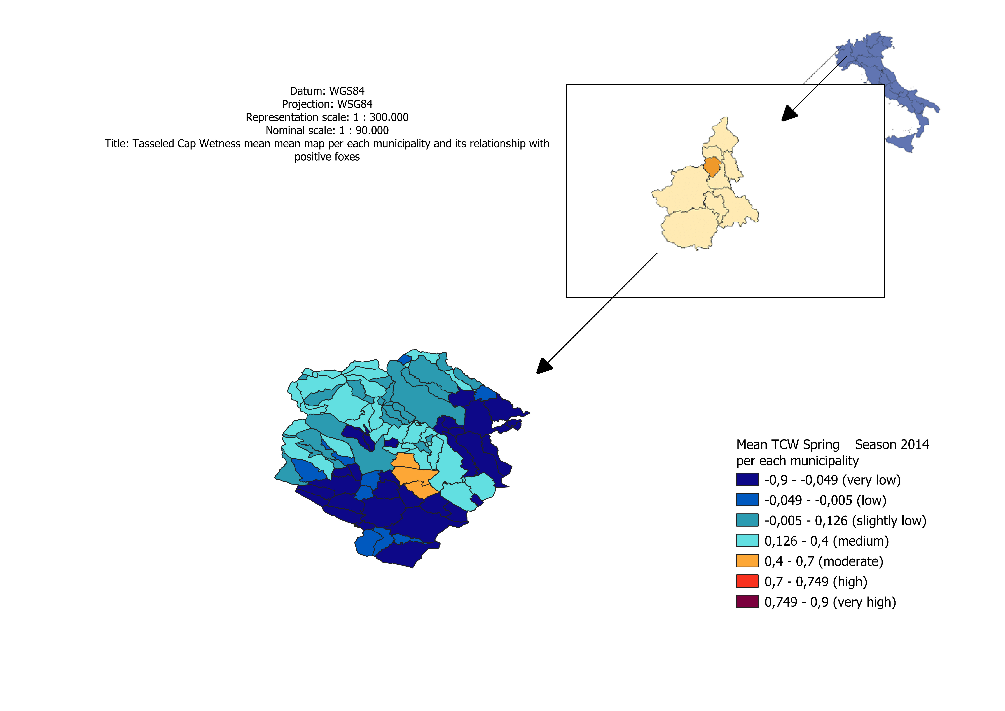


Figure C. Local spatial autocorrelation indicator (LISA) map for Tasseled Cap Wetness (TCW) for each municipality in Piedmont (province of Biella) in winter 2014.


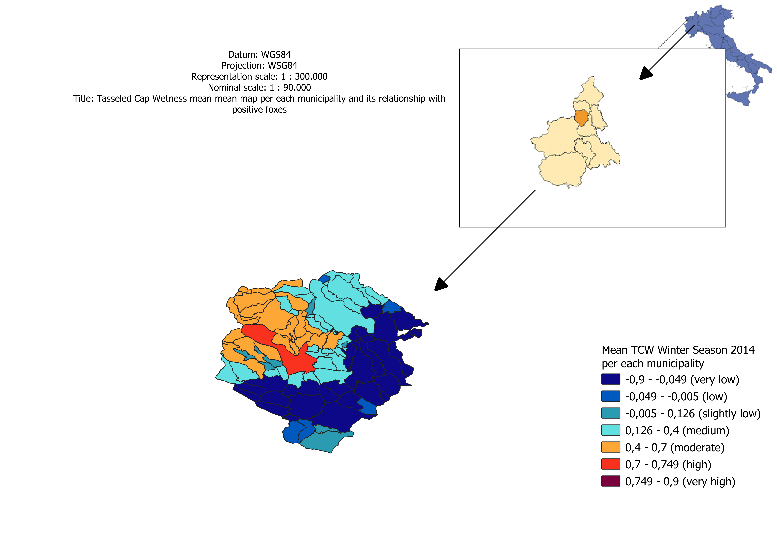


Figure D. Local spatial autocorrelation indicator (LISA) map for Tasseled Cap Wetness (TCW) for each municipality in Piedmont (province of Cuneo) in winter 2021.


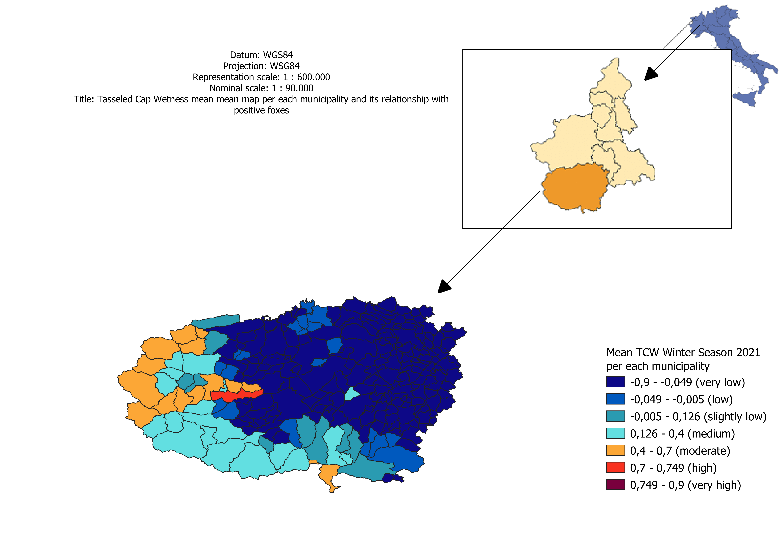


Figure E. Local spatial autocorrelation indicator (LISA) map for Tasseled Cap Wetness (TCW) for each municipality in Aosta Valley in winter 2022.


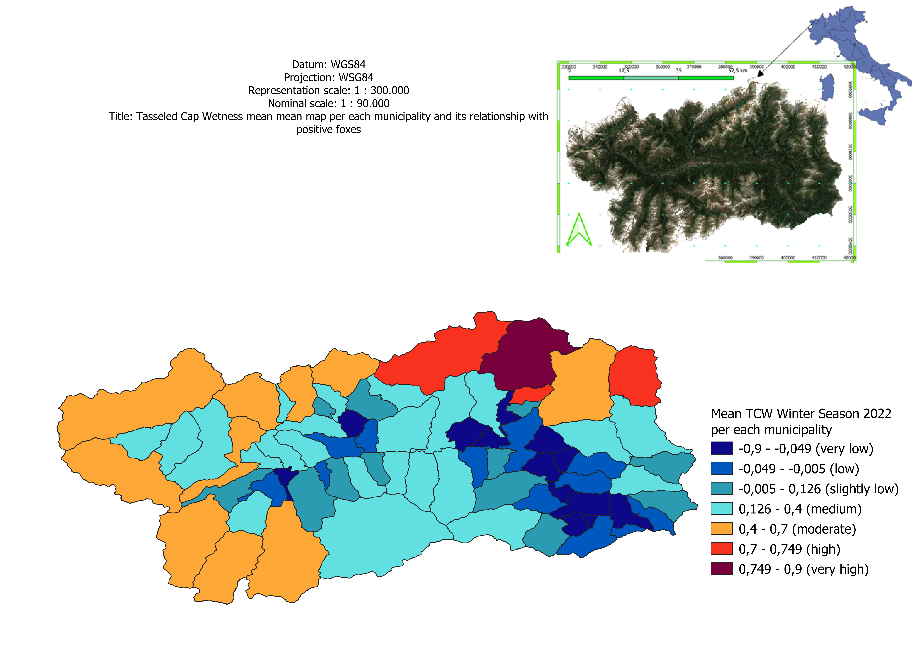


**Statistics analysis**

**CHI-2 TEST**

**Sex (Male vs Female)**

Chi squared

Degrees freedom: 1

Chi2: 0,010606 p (no assoc.): 0,91798

Monte Carlo p : 1

Fisher´s exact

p (no assoc.): 1

Other statistics

Cramer´s V : 0,0096453 Contingency C : 0,0096448

**Animal area (province)**

Chi squared

Degrees freedom: 2

Chi2: 0,12668 p (no assoc.): 0,93863

Monte Carlo p : 1

Fisher´s exact

p (no assoc.): 0,86789

Other statistics

Cramer´s V : 0,037941 Contingency C : 0,037913

**Age**

Chi squared

Degrees freedom: 1

Chi2: 0,046491 p (no assoc.): 0,82929

Monte Carlo p : 1

Fisher´s exact

p (no assoc.): 1

Other statistics

Cramer´s V : 0,020943 Contingency C : 0,020938

**Years period**

Chi squared

Degrees freedom: 1

Chi2: 2,249 p (no assoc.): 0,1337

Monte Carlo p : 0,2617

Fisher´s exact

p (no assoc.): 0,15125

Other statistics

Cramer´s V : 0,14046 Contingency C : 0,13909

**Seasons (Fall-Winter and Spring-Summer)**

Chi squared

Degrees freedom: 1

Chi2: 3,6137 p (no assoc.): 0,057304

Monte Carlo p : 0,0767

Fisher´s exact

p (no assoc.): 0,078172

Other statistics

Cramer´s V : 0,17804 Contingency C : 0,17529
